# Effect of Neuroligin1 and Neurexin1 on the Colonic Motility in a Mouse Model of Neuronal Intestinal Dysplasia

**DOI:** 10.1155/2020/9818652

**Published:** 2020-01-04

**Authors:** Dongming Wang, Ni Gao, Tingting Zhou, Qiangye Zhang, Jian Wang, Aiwu Li

**Affiliations:** Department of Pediatric Surgery, Qilu Hospital, Shandong University, China

## Abstract

**Aim:**

To investigate the expressions of neuroligin1 (NL1) and neurexin1 (NX1) in a mouse model of neuronal intestinal dysplasia (Tlx2^−/−^ mice) and to explore their effects on colonic motility.

**Methods:**

Immunohistochemistry staining was employed to explore the histological appearances of NL1, NX1, the presynaptic marker of glutamatergic synapses VGLUT1, and the subunit of NMDA receptors of NR1 in the colon of mice with or without Tlx2 mutation. Western blotting and qRT-PCR were performed to detect their relative expressions in the colon. Colonic motility was measured by a glass bead technique. Then, the Tlx2^−/−^ mice were intervened by Huperzine A. Variations on expressions of NL1, NX1, VGLUT1, and NR1 and variations on colonic motility were measured. Additionally, serum concentrations of Glu were measured by ELISA.

**Results:**

Immunohistochemistry staining reveals that NL1, NX1, VGLUT1, and NR1 were mainly concentrated in the myenteric plexus of ENS. Compared to those in WT and Tlx2^+/-^ mice, expressions of NL1 and NX1 in colon of Tlx2^−/−^ mice were upregulated with increased VGLUT1 and NR1 abundances and impaired colonic motility (*P* < 0.05). After intervention, the upregulated expressions of NL1 and NX1 were decreased with a correlated reduce of VGLUT1 and NR1 and a recovery of the impaired colonic motility (*P* < 0.05). After intervention, the upregulated expressions of NL1 and NX1 were decreased with a correlated reduce of VGLUT1 and NR1 and a recovery of the impaired colonic motility (*P* < 0.05). After intervention, the upregulated expressions of NL1 and NX1 were decreased with a correlated reduce of VGLUT1 and NR1 and a recovery of the impaired colonic motility (

**Conclusion:**

NL1 and NX1 are closely related to the colonic motility through their effects of targeting the formation of glutamatergic synapses and may be involved in the pathogenesis of NID. The variations of serum Glu seem to be a potential and less painful auxiliary measure for colonic motility and NID.

## 1. Introduction

Neuronal intestinal dysplasia (NID) is a clinical condition that resembles Hirschsprung's disease (HSCR) and is clinically characterized by seriously impaired colonic motility, such as severe constipation or intestinal obstruction [1, 2]. Different from HSCR in histologically observation, absence of ganglion cells in the myenteric plexus, patients with NID show hyperplasia of the submucous and myenteric plexus with increase in AChE-positive nerve fibers [3]. Although pathological innervation patterns have been described in the colon of patients with NID, it remains controversial as to whether there is a causal relationship between histological findings and its seriously impaired colonic motility. Furthermore, due to striking similarities among clinical features with HSCR, a definitive diagnosis of NID can be made only on histological analysis of rectal biopsies [1, 4]. Therefore, a further research of the pathogenesis of NID and a more convenient and less painful diagnostic method are still needed.

Synapses, connecting neurons into overlapping and interdigitated neural circuits, are fundamental units of the nervous system and are vital to the functional neural network. Neurexins represent a family of presynaptic neuronal cell adhesion proteins and form transsynaptic complexes with neuroligins, the most well-known postsynaptic ligand of neurexins, by protein domains or heparan sulfate (HS) [5, 6]. Interactions between neurexins and neuroligins orchestrate the events of formation and stabilization of both excitatory and inhibitory synapses and are central to a shared genetic risk pathway in autism and schizophrenia in the central nervous system (CNS) [7, 8]. Proper functioning of gastrointestinal (GI) motility also critically depends on a delicate coordination of excitatory and inhibitory enteric impulses within the enteric nervous system (ENS) [9]. Although less researches have been conducted in ENS, previous studies have shown that neurexins and neuroligins also expressed in ganglion cells of myenteric plexus [10–12]. So, a hypothesis to account for the histological findings and seriously impaired colonic motility of NID was proposed that interactions of neurexins and neuroligins may be correlated with the colonic motility.

Homozygous mutant mice of Tlx2 (Tlx2^−/−^) display an intestinal phenotype with impaired colonic motility and have been verified strongly resembling NID [13, 14]. To verify this hypothesis, the Tlx2^−/−^ mice were chosen as the animal model. Vesicular glutamate transporter-1 (VGLUT1), a presynaptic molecule transporting Glu into synaptic vesicles and assisting its release at presynaptic terminals, and NR1 subunit of NMDA receptors (NR1), both of which can be clustered by interaction of neuroligin1 (NL1) and neurexin1 (NX1) during synaptogenesis [15, 16], were chosen as markers of glutamatergic synapses. Further researches were employed to explore the pathogenesis of NID through the effects of NL1 and NX1 on colonic motility.

## 2. Materials and Methods

### 2.1. Animal and Sample Preparation

Our study was approved by the ethics committee of Qilu Hospital, Shandong University (No. 12025). The mice were treated under the animal use guidelines of Institutional Animal Care and Use Committee (IACUC) of Qilu Hospital, Shandong University. 173 bp region of the nucleotide sequence at the second exon of the Tlx2 gene was knocked out through the CRISPR/Cas9 gene-targeting technique, and Tlx2^+/-^ mice were generated by Genechem (Shanghai, China). Tlx2^+/-^ mice were interbred to obtain Tlx2^−/−^ mice. Three weeks after being born, the mutant offspring were numbered and segments of tails were harvested for genotyping. At age of 8 weeks or 1 day after drug intervention, colonic motilities were measured. After applying anaesthesia via pentobarbital, blood samples and segments of the full-thickness distal colon were harvested. Then, serums were harvested through centrifugation after clotting. Serums and colon tissues were stored at −80°C for further assay.

### 2.2. Genotyping

Tlx2 genotypes were determined by Southern blot. The genomic DNA was isolated from the tails of the mutant offspring by a Mouse Tail Genomic DNA Kit (CWBio, Beijing, China). Primers of Tlx2 are shown in Table 1, and the reaction solution consisted of 25 *μ*l 2× Es Taq MasterMix (CWBio, Beijing, China), 2 *μ*l forward primer (10 *μ*mol/l), 2 *μ*l reverse primer (10 *μ*mol/l), 2.5 *μ*l DMSO (Solarbio, Beijing, China), 16 *μ*l double steaming water, and 2.5 *μ*l genomic DNA was prepared. Then the genomic DNA was amplified by PCR and separated on 1% agarose gel. The image was taken by a BIO-RAD gel documentation system (Bio-Rad, CA, USA).

### 2.3. Drug Intervention

Tlx2^−/−^ mice were intervened by Huperzine A, a lycopodium alkaloid isolated from the herb Huperzia serrata affecting the expression of NX1 [17]. Huperzine A (Beijing Institute of Pharmacology and Toxicology, Beijing, China) was dissolved in 0.1 N HCl at 5 mg/ml as stock solution and diluted before usage with physiological saline. Tlx2^−/−^ mice were divided into 5 groups randomly. Huperzine A was given to Tlx2^−/−^ mice at a dose of 0.1 mg/Kg for 8 weeks by intragastric gavage (group HIG) at 12.5 times dilution and retention-enema (group HRE) at 25 times dilution. At the same time, a same dose of physiological saline was given also through intragastric gavage (group SIG) and retention-enema (group SRE). The Tlx2^−/−^ mice without any intervention were the CON group.

### 2.4. Colonic Motility Measuring

Colonic motilities of mice were measured by a glass bead technique. Wrap a mouse in a towel and keep the mouse calm. Strike gently around the mouse for 5 min to stimulate the mouse to pass fecal pellets. A glass bead (2.5 mm diameter) is pushed slowly into the colon 2 cm through the anus with a glass rod (2.5 mm diameter and marked 2 cm). After checking that the bead is not stuck beside the glass rod, the rod is removed slowly. Once the rod is removed, start the timer until the bead is expelled out.

### 2.5. Immunohistochemistry Staining

Immunohistochemistry staining was employed to detect the histological appearance of NL1, NX1, NR1, and VGLUT1. The paraffin sections were dewaxed in xylene and graded alcohols. Antigens were unmasked by microwaving in 0.01 M citrate buffer, and endogenous peroxidases were ablated by 3% H_2_O_2_. After being rinsed with 0.1 M PBS, the colon sections were blocked with goat serum for 0.5 h at 37°C and incubated in primary antibodies (Table 2) at 4°C overnight. Then the colon sections were incubated with secondary antibodies at 37°C for 0.5 h and stained by DAB (Beyotime, Shanghai, China). After dehydration with graded alcohols and xylene, the colon sections were coverslipped and pictured.

### 2.6. Western Blot Analysis

Western blot analysis was applied for investigating the relative abundances of NL1, NX1, NR1, and VGLUT1 at protein level. Total proteins were isolated from full-thickness colon specimens by the Minute™ Total Protein Extraction Kit (Invent, MN, USA). After, concentrations measuring 20 *μ*g of protein were separated on 7.5% SDS-PAGE gel and electroblotted onto PVDF membranes. After being blocked by 5% BSA, the PVDF membranes were incubated with primary antibodies (Table 2) at 4°C overnight. Then, the membranes were rinsed by 0.1 M TBST and incubated with secondary antibodies (Table 2) for 1 h at RT. After being rinsed by TBST, the membranes were detected by an ECL kit (Millipore, MA, USA), and the gray values were calculated finally.

### 2.7. qPCR Assay

The qPCR assay was applied for investigating the relative expressions of NL1, NX1, NR1, and VGLUT1 at mRNA level. Total RNA of each specimen was isolated by a MiniBEST Universal Extraction Kit (TaKaRa, Shiga, Japan). After assessing the concentration, the cDNA was synthesized with an amount of 1 *μ*g RNA according the instructions of the PrimeScript™ RT Reagent Kit (TaKaRa, Shiga, Japan). Then, reaction solutions were prepared using a UltraSYBR Mixture (CWBio, Beijing, China), and qPCR reactions were performed at a Roche LightCycler 480 system. Detailed information of primers is shown in Table 1. The Ct values were measured and 2^−*Δ*ΔCt^ of each group was calculated for statistical analysis.

### 2.8. ELISA Assay

Serum specimens stored at −80°C were thawed on ice for preparation. The Glu ELISA Kit (Xinqidi, Wuhan, China) was chosen to detect the levels of Glu. Serum specimens were added to 96-well detection plates (100 *μ*l per well). After the reaction, the OD values were measured at 450 nm and the actual serum concentrations of Glu were calculated according to the standard curve.

### 2.9. Statistical Analyses

All data were analyzed by GraphPad Prism® 7.0 software and shown as mean ± SD. One-way ANOVA and Tukey's test were used for multiple comparisons, and *P* values less than 0.05 were considered to be statistically significant.

## 3. Results

### 3.1. Genotyping and Autopsy

**Figure 1:**
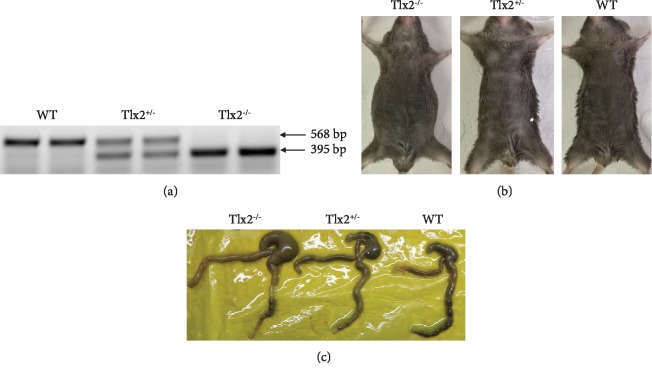
Genotyping and autopsy of mice: (a) 173 bp nucleotide sequence of Tlx2 gene was knocked out in Tlx2^−/−^ mice; (b) distended abdomens were often seen in Tlx2^−/−^ mice; (c) gross anatomy showed no abnormality in the Tlx2^+/-^ mice. The appendix, cecum, distal ileum, and proximal colon of the Tlx2^−/−^ mice were dilated compared to the WT and Tlx2^+/-^ mice.

Tlx2^+/-^ mice showed normal up to 1.5 years of age. Tlx2^+/-^ mice were interbred, and their offspring were genotyped by the Southern blot analysis (Figure 1(a)). The wild-type allele was shown as a 568 bp fragment, and the mutant allele was shown as a 395 bp fragment. The 173 bp nucleotide sequence of Tlx2 gene was knocked out in the Tlx2^−/−^ mice. Tlx2^−/−^ mice often exhibited retarded growth with a lower weight and distended abdomens (Figure 1(b)), as previously reported [13, 14]. 26% (33/127) of the Tlx2^−/−^ mice died up to 8 weeks after birth. The Tlx2^−/−^, Tlx2^+/-^, and WT mice were anatomized and intestinal tracts were dissected. Gross anatomy showed no abnormality in the Tlx2^+/-^ mice compared to WT mice. The appendix, cecum, distal ileum, and proximal colon of the Tlx2^−/−^ mice were dilated, and the distal colon of the Tlx2^−/−^ mice appeared constricted compared to the WT and Tlx2^+/-^ mice (Figure 1(c)).

### 3.2. Upregulated Expressions of NL1 and NX1 and Impaired Colonic Motility in Tlx2^+/-^ Mice

**Figure 2:**
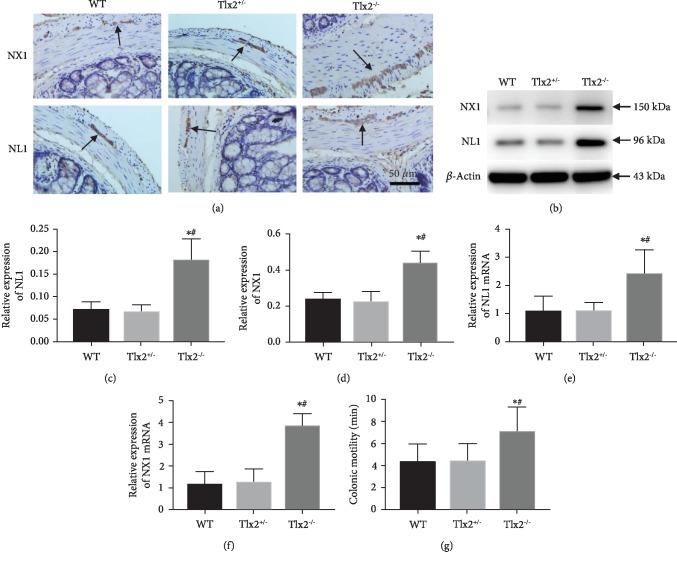
Upregulated expressions of NL1 and NX1 and impaired colonic motility in Tlx2^−/−^ mice. Immunohistochemistry staining (a) revealed that positive cells of NL1 and NX1 in the distal colon were ganglion cells of the myenteric plexus and the myenteric plexus was hyperplasia. Representative blots (b), comparisons of relative gray values (c, d) in Western blot analysis and comparisons of mRNA relative expressions in qPCR assay (e, f) indicated that expressions of NL1 and NX1 in the Tlx2^−/−^ mice were upregulated compared to that in the WT and Tlx2^+/-^ mice. The longer expelling time of the bead in the Tlx2^−/−^ mice indicated an impaired colonic motility (g). ^∗^*P* < 0.05 versus WT; ^#^*P* < 0.05 versus Tlx2^+/-^.

Histological appearances of NL1 and NX1 in distal colon by immunohistochemistry staining (Figure 2(a)) showed that positive-stained cells of both NL1 and NX1 were concentrated mostly in ganglion cells of myenteric plexus, which was consistent with the previous finding [10, 18]. Hyperplasia of the myenteric plexus was also observed as previously reported [13, 14]. Representative blots (Figure 2(b)) and comparisons of relative gray values (Figures 2(c) and 2(d)) indicated that the relative expressions of protein NL1 and NX1 in the Tlx2^−/−^ mice (0.180 ± 0.049, 0.435 ± 0.067) were upregulated compared to those in the WT (0.069 ± 0.018, 0.234 ± 0.042) and Tlx2^+/-^ (0.062 ± 0.020, 0.234 ± 0.042) mice (*P* < 0.05). qPCR (Figures 2(e) and 2(f)) shown a similar trend that NL1 and NX1 mRNA were higher in the Tlx2^−/−^ mice (2.423 ± 0.872, 3.839 ± 0.613) compared to those in the WT (1.097 ± 0.540, 1.133 ± 0.610) and Tlx2^+/-^ (1.095 ± 0.310, 1.242 ± 0.624) mice (*P* < 0.05). The glass bead technique was used to detect the colonic motility of mice (Figure 2(g)). The time of bead being expelled out in the Tlx2^−/−^ mice (7.053 ± 2.278 min) was longer compared to that in the WT and Tlx2^+/-^ mice (4.357 ± 1.560, 4.468 ± 1.556 min, *P* < 0.05). The longer expelling time indicated an impaired colonic motility in the Tlx2^−/−^ mice. Comparisons between the Tlx2^+/-^ and WT mice expressed no statistical difference.

### 3.3. Increase of Glutamatergic Synapses in Tlx2^+/-^ Mice

**Figure 3:**
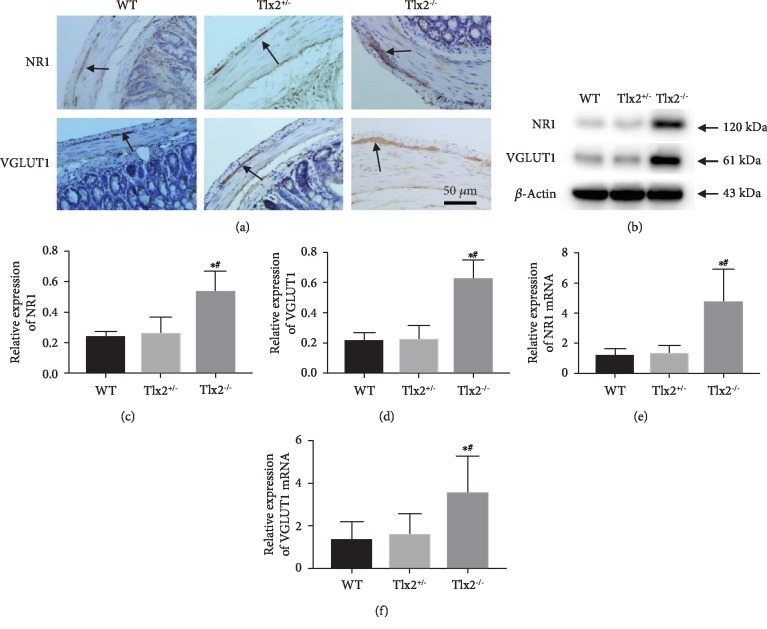
Increase of glutamatergic synapses in Tlx2^+/-^ mice: immunohistochemistry staining (a) showed that glutamatergic synapses marked by NR1 and VGLUT1 existed and mainly concentrated in the myenteric plexus of the distal colon of mice; Western blot (b–d) and qPCR (e, f) indicated that expressions of NR1 and VGLUT1 in the Tlx2^−/−^ mice were higher compared to those in the WT and Tlx2^+/-^ mice, which indicated the increase of glutamatergic synapses in the Tlx2^−/−^ mice colon (^∗^*P* < 0.05 versus WT, ^#^*P* < 0.05 versus Tlx2^+/-^).

NR1 was a subunit of NMDA receptors of glutamatergic synapses and VGLUT1 was a presynaptic maker of glutamatergic synapses. Immunohistochemistry staining (Figure 3(a)) showed that NR1 and VGLUT1 were expressed mainly in the distal colon myenteric plexus of mice and slightly in the mucosa and submucosa, which indicated the existence of glutamatergic synapses. Western blots (Figures 3(b)–3(d)) revealed that the relative abundance of NR1 and VGLUT1 in the Tlx2^−/−^ mice (0.533 ± 0.134, 0.621 ± 0.129) was higher compared to that in the WT mice (0.235 ± 0.040, 0.210 ± 0.061) and the Tlx2^+/-^ mice (0.257 ± 0.109, 0.217 ± 0.100) (*P* < 0.05). The qPCR assay (Figures 3(e) and 3(f)) confirmed the trend at mRNA level that the NR1 and VGLUT1 mRNA in the Tlx2^−/−^ mice were higher (4.708 ± 2.176, 3.520 ± 1.717) compared to those in the WT (1.101 ± 0.498, 1.272 ± 0.893) and Tlx2^+/-^ (1.268 ± 0.588, 1.561 ± 0.978) mice (*P* < 0.05). The higher expressions of NR1 and VGLUT1 indicated the increase of glutamatergic synapses in the Tlx2^−/−^ mice colon.

### 3.4. Decrease of the Upregulated Expressions of NL1 and NX1 and Correlated Recovery of the Impaired Colonic Motility after Intervention

**Figure 4:**
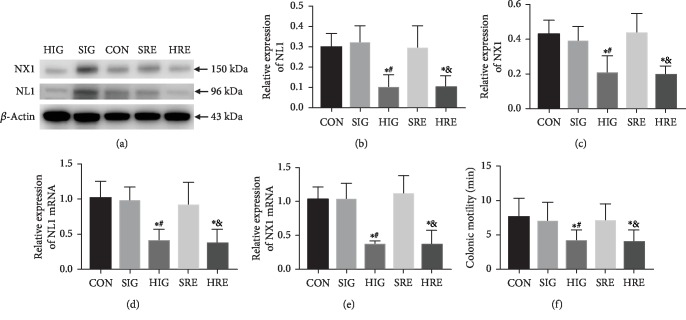
Decrease of the upregulated expressions of NL1 and NX1 and correlated recovery of the impaired colonic motility after intervention. Huperzine A was given to the Tlx2^−/−^ mice by intragastric gavage and retention-enema for 8 weeks. Western blots (a–c) show that the upregulated expressions of protein NL1 and NX1 decreased after intervention in groups HIG and HRE. qPCR assay (d, e) confirmed the decreased expressions of NL1 and NX1. Shorten expelling times of bead in group HIG and HRE represented a correlated recovery of the impaired colonic motility (f) (^∗^*P* < 0.05 versus CON, ^#^*P* < 0.05 versus SIG, ^&^*P* < 0.05 versus SRE).

The expressions of NL1 and NX1 and the colonic motility after intervention are shown in Figure 4. Expressions of protein NL1 and NX1 in group HIG (0.100 ± 0.064, 0.203 ± 0.102) and HRE (0.103 ± 0.055, 0.193 ± 0.050) decreased significantly compared to those in group CON (0.300 ± 0.066, 0.425 ± 0.083), SIG (0.317 ± 0.088, 0.383 ± 0.093), and SRE (0.292 ± 0.112, 0.432 ± 0.111, *P* < 0.05). The qPCR assay confirmed the decreased expressions of NL1 and NX1. Expressions of NL1 and NX1 mRNA in group HIG (0.411 ± 0.161, 0.360 ± 0.051) and HRE (0.375 ± 0.202, 0.350 ± 0.223) were reduced significantly compared to those in group CON (1.022 ± 0.242, 1.016 ± 0.203), SIG (0.982 ± 0.200, 1.010 ± 0.256), and SRE (0.915 ± 0.330, 1.106 ± 0.267, *P* < 0.05). Colonic motility measuring revealed a correlated recovery of the impaired colonic motility. Expelling times of the bead in groups HIG and HRE (4.068 ± 1.653, 3.989 ± 1.650 min) were shorter compared to those in the groups CON, SIG, and SRE (7.600 ± 2.792, 6.977 ± 2.785, 7.012 ± 2.502 min, *P* < 0.05). The physiological saline cannot affect the colonic motility.

### 3.5. Correlated Reduction of Glutamatergic Synapses after Intervention

**Figure 5:**
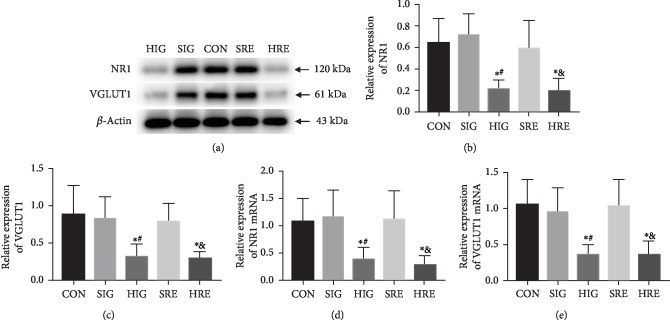
Correlated reduction of glutamatergic synapses after intervention. Representative blots (a) and comparisons of relative gray values (b, c) revealed decreased expressions of protein NR1 and VGLUT1 in groups HIG and HRE after intervention. qPCR (d, e) confirmed the variations of NR1 and VGLUT1 at mRNA level. The reduction of the expressions of NR1 and VGLUT1 also reflected the reduction of glutamatergic synapses after intervention (^∗^*P* < 0.05 versus CON, ^#^*P* < 0.05 versus SIG, ^&^*P* < 0.05 versus SRE).

The variations of NR1 and VGLUT1 after intervention are shown in Figure 5. Expressions of protein NR1 and VGLUT1 in groups HIG (0.216 ± 0.083, 0.255 ± 0.113) and HRE (0.195 ± 0.115, 0.245 ± 0.098) decreased significantly compared to those in groups CON (0.462 ± 0.220, 0.581 ± 0.110), SIG (0.708 ± 0.205, 0.630 ± 0.156), and SRE (0.586 ± 0.259, 0.656 ± 0.186, *P* < 0.05). The qPCR assay confirmed this variations in mRNA level. NR1 and VGLUT1 mRNA in groups HIG (0.378 ± 0.227, 0.358 ± 0.139) and HRE (0.282 ± 0.164, 0.356 ± 0.193) was reduced compared to that in groups CON (1.068 ± 0.430, 1.050 ± 0.360), SIG (1.156 ± 0.494, 0.951 ± 0.338), and SRE (1.107 ± 0.526, 1.037 ± 0.371, *P* < 0.05). The reduction of the expressions of NR1 and VGLUT1 also reflected the reduction of glutamatergic synapses.

### 3.6. Correlated Variations of Serum Concentrations of Glu

**Figure 6:**
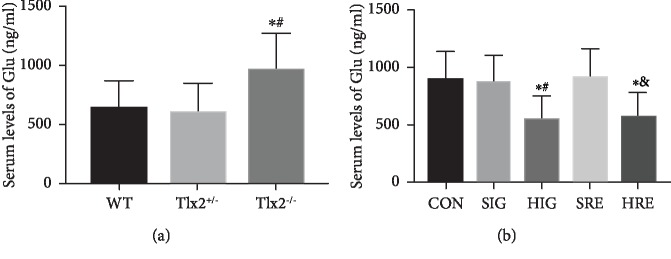
Correlated variations of serum concentrations of Glu: (a) the serum level of Glu in the Tlx2^−/−^ mice was higher compared to those in the WT and Tlx2^+/-^ mice (^∗^*P* < 0.05 versus WT, ^#^*P* < 0.05 versus Tlx2^+/-^); (b) after intervention, the serum levels of Glu in groups HIG and HRE decreased compared to those in groups CON, SIG, and SRE (^∗^*P* < 0.05 versus CON, ^#^*P* < 0.05 versus SIG, ^&^*P* < 0.05 versus SRE).

Variations of serum concentrations of Glu measured by ELISA were in concordance with the variations of glutamatergic synapses marked by NR1 and VGLUT1 and the variations of colonic motility. The serum level of Glu in the Tlx2^−/−^ mice (967.8 ± 305.1 ng/ml) was higher compared to that in the WT and Tlx2^+/-^ mice (642.5 ± 234.0, 602.1 ± 259.4 ng/ml, *P* < 0.05; Figure 6(a)). After intervention, the serum levels of Glu in groups HIG and HRE (545.1 ± 200.6, 568.5 ± 213.2 ng/ml) decreased compared to those in groups CON, SIG, and SRE (893.0 ± 248.7, 866.9 ± 238.8, 913.2 ± 252.7 ng/ml, *P* < 0.05; Figure 6(b)).

## 4. Discussion

The ENS arises from the enteric neural crest cells [19, 20]. Failures in development of ENS and consequent impairments will cause severe intestinal motor disorders, such as the complicated disorder NID. Many investigators have raised doubts about the existence of NID as a distinct histopathologic entity and suggested that the pathologic changes seen in IND may be a secondary phenomenon induced by intestinal obstruction and inflammatory disease [1, 21]. The Tlx2^−/−^ mice is a strong evidence that NID is a real entity. In the present study, the gene Tlx2 was knocked out. Then, hyperplasia of the myenteric plexus was observed in the distal colon. The malformation of ENS may bring about the constricted distal colon shown in a gross anatomy. The dilation of the appendix, cecum, distal ileum, and proximal colon may be a secondary variation induced by the colon obstruction. As a result, a distended abdomen and the impaired colonic motility of the Tlx2 mice were not amazing. Those findings confirmed the candidate of the Tlx2^−/−^ mice resembling an animal model of NID.

Intestinal motility causes proper anterograde propulsion of luminal contents through well-coordinated contraction and relaxation of the gut smooth muscle [22]. The longer expelling time of the bead in live Tlx2^−/−^ mice indicated an impaired colonic motility. At the same time, the expressions of NL1 and NX1 in the ENS of the distal colon of the Tlx2^−/−^ mice were upregulated. Tlx2 is an orphan homeobox gene specifically expressing in tissues derived from neural crest cells and encodes homeodomain, known to bind specific DNA sequences to regulate the expression of downstream genes in the developing ENS [23, 24]. So, there may be a direct or an indirect interaction among NL1, NX1, and Tlx2. The malfunction of Tlx2 may lead to the upregulated expressions of NL1 and NX1. But this needs further research to verify. After intervention, the upregulated expressions of LN1 and NX1 decreased, accompanied by a recovery of the impaired colonic motility. The correlations between expressions of NL1 and NX1 and variations of colonic motility verified the prediction that NL1 and NX1 were closely related to the colonic motility and may be involved in the pathogenesis of NID, which was an interpretation that the impaired colonic motility of Tlx2^−/−^ mice may have resulted from hyperplasia of the myenteric plexus in the distal colon as shown in this paper.

As a major excitatory neurotransmitter in CNS, Glu plays a fundamental role in the physiological condition [25]. Despite contentious topics in the literature and that exact pathways have not yet been fully elucidated, increasing evidences suggest that glutamate may also participate in the regulation of the GI motility, as well as in the brain-gut axis [25–27]. NL1 is localized to glutamatergic synapses and can bind to NX1 to target the formation and differentiation of glutamatergic synapses by clustering synaptic proteins such as NR1 and VGLUT1 [15, 16]. The finding that NR1 and VGLUT1 are expressed in the myenteric plexus of the mice colon indicated the existence of glutamatergic synapses in the ENS.

The variations of NR1 and VGLUT1 were accompanied by the variations of NL1 and NX1. The upregulated expressions of LN1 and NX1 in Tlx2^−/−^ mice can lead to the increase of glutamatergic synapses of ENS, which was verified by the correlated increase of NR1 and VGLUT1. Proper functioning of colon motility critically depends on a delicate coordination of excitatory and inhibitory enteric impulses [28]. The increase of glutamatergic synapses will generate ascending excitatory synaptic impulses, which will lead to the uncoordinated contraction of smooth muscle cells. The strengthened contraction, which was observed in distal colon of the Tlx2 mice, will finally cause colonic dysmotility as a consequence. Likewise, after the upregulated expressions of LN1 and NX1 decreased by the intervention, the recovery of the glutamatergic synapses will improve the impaired colonic motility. These indicated that NL1 and NX1 may affect the colonic motility through glutamatergic synapses.

Due to the similar clinical symptoms between NID and HSCR, definitive diagnosis still depends on histopathological analysis of rectal biopsies nowadays [1]. Rectal biopsy is a skillful and delicate method which only gives excellent results if it is carried out by a very competent surgeon [29]. A more convenient and less painful diagnostic method is still needed. In this study, variations of serum Glu measured by ELISA were in concordance with the variations of glutamatergic synapses and colonic motility. The ascending serum Glu in Tlx2^−/−^ mice may reflect its increase of glutamatergic synapses and the consequent colonic dysmotility in some degree and vice versa. The variations of serum Glu concentrations seem to be a potential and less painful auxiliary measure for colonic motility and NID.

As shown above, we concluded that NL1 and NX1 were closely related to the colonic motility and may be involved in the pathogenesis of NID. The over-expressions of NL1 and NX1 in NID mice colon, which generate the increases glutamatergic synapses of ENS, may lead to colonic dysmotility consequently. Furthermore, the variations of serum Glu concentrations seem to be a potential and less painful auxiliary measure for colonic motility and NID.

## Figures and Tables

**Table 1:** Detailed information of primers.

Primer	F sequences (5′-3′)	R sequences (5′-3′)
Tlx2	TTGATGAGGCTTCTGTGGTT	AAGAGCGACGAGTTGTGC
NL1	GACCACCAACGACCTAAC	TCCGAAGAACCACCTCAT
NX1	GCAAGCCAAGACATCAGA	GCCATCCAGCATCTCAATA
NR1	GCACACTGGACTCATTCA	TCCTCGCTGTTCACCTTA
VGLUT1	ATGAGCGAGGAGGAGTGT	AGGTGTATGGAGTGGAAGT
*β*-Actin	CCACCATGTACCCAGGCATT	ACGCAGCTCAGTAACAGTCC

**Table 2:** Detailed information of antibodies.

Antigen	Description	Dilution	Source
NL1	Mouse anti-Mouse	1 : 1000	abcam, MA, USA
NX1	Goat anti-Mouse	1 : 1000	abcam, MA, USA
NR1	Rabbit anti-Mouse	1 : 1000	Affinity, Changzhou, China
VGLUT1	Rabbit anti-Mouse	1 : 2000	SYSY, Gottingen, Germany
*β*-Actin	Mouse anti-Mouse	1 : 1000	Beyotime, Shanghai, China
IgG(H+L)	Goat anti-Mouse	1 : 5000	Beyotime, Shanghai, China
IgG(H+L)	Goat anti-Rabbit	1 : 5000	Beyotime, Shanghai, China
IgG(H+L)	Donkey anti-Goat	1 : 5000	Beyotime, Shanghai, China

## Data Availability

The data used to support the findings of this study are available from the corresponding author upon request.
